# Supplementary material for: Detailed molecular and epigenetic characterization of the pig IPEC-J2 and chicken SL-29 cell lines
Source: iScience. 2023 Feb 20;26(3):106252. doi: 10.1016/j.isci.2023.106252 (PMC10018572; doi:10.1016/j.isci.2023.106252)
Supplement: Data S2. Complete homer output for identified motifs in Chicken SL-29, related to Tables 5 and 6 — Homer motif analysis results for histone modifications H3K4me1, H3K4me3, H3K27ac, enhancers, and ATAC-seq of chicken SL-29 cell line. Parameters for possible false positives is as mentioned earlier for S5. [file mmc3.zip › Data_S2/S6/Chicken_SL_29/peak_files_ATAC/homerResults/motif1.similar.html]

motif1

## Information for motif1

C
G
T
A
G
A
T
C
A
C
T
G
A
T
G
C
C
T
A
G
C
A
G
T
T
C
A
G
C
G
T
A
G
A
C
T
G
C
A
T
  
Reverse Opposite:  

C
G
T
A
C
G
T
A
A
C
G
T
A
G
T
C
G
T
C
A
G
A
T
C
T
A
C
G
T
G
A
C
C
T
A
G
A
C
G
T
  

|  |  |
| --- | --- |
| p-value: | 1e-1706 |
| log p-value: | -3.930e+03 |
| Information Content per bp: | 1.732 |
| Number of Target Sequences with motif | 1513.0 |
| Percentage of Target Sequences with motif | 1.74% |
| Number of Background Sequences with motif | 43.1 |
| Percentage of Background Sequences with motif | 0.05% |
| Average Position of motif in Targets | 146.9 +/- 84.5bp |
| Average Position of motif in Background | 146.7 +/- 69.9bp |
| Strand Bias (log2 ratio + to - strand density) | -0.0 |
| Multiplicity (# of sites on avg that occur together) | 1.54 |
| Motif File: | file (matrix) reverse opposite |

### Similar de novo motifs found

|  |  |  |  |  |  |  |  |
| --- | --- | --- | --- | --- | --- | --- | --- |
| Rank | Match Score | Redundant Motif | P-value | log P-value | % of Targets | % of Background | Motif file |
| 1 | 0.611 | A C T G C A G T A C T G C T G A A C G T A C G T A G C T A C G T A G T C A C T G | 1e-1573 | -3622.325842 | 1.44% | 0.03% | motif file (matrix) |
| 2 | 0.765 | C T A G A G T C C T A G C G A T T C A G C G T A A G C T C G A T A C G T A C G T | 1e-1519 | -3497.664697 | 1.90% | 0.09% | motif file (matrix) |
| 3 | 0.800 | C G T A C G T A A C G T G T A C G T A C G A T C G T A C G A T C C T G A G A C T | 1e-467 | -1075.384767 | 2.14% | 0.58% | motif file (matrix) |
| 4 | 0.683 | T C A G C T G A T G C A C G T A G T C A G C A T A G T C C G T A A G T C T A C G | 1e-401 | -924.294993 | 3.95% | 1.74% | motif file (matrix) |
| 5 | 0.608 | A C G T A C T G C G T A A C G T C G A T A C G T A C G T A T G C | 1e-232 | -534.345277 | 4.61% | 2.66% | motif file (matrix) |
